# CASK Mediates Oxidative Stress-Induced Microglial Apoptosis-Inducing Factor-Independent Parthanatos Cell Death via Promoting PARP-1 Hyperactivation and Mitochondrial Dysfunction

**DOI:** 10.3390/antiox13030343

**Published:** 2024-03-13

**Authors:** Keith Jun Hao Cheong, Duen-Yi Huang, Ponarulselvam Sekar, Rou Jhen Chen, Irene Han-Juo Cheng, Chi-Ming Chan, Yuan-Shen Chen, Wan-Wan Lin

**Affiliations:** 1 Department of Pharmacology, College of Medicine, National Taiwan University, Taipei 100233, Taiwan; r10443021@ntu.edu.tw (K.J.H.C.); tyhuang123@ntu.edu.tw (D.-Y.H.); r08443012@ntu.edu.tw (R.J.C.); 2 Graduate Institute of Medical Sciences, Taipei Medical University, Taipei 110301, Taiwan; d119101011@tmu.edu.tw; 3 Institute of Brain Science, National Yang Ming Chiao Tung University, Taipei 112304, Taiwan; ihjcheng@nycu.edu.tw; 4 Department of Ophthalmology, Cardinal Tien Hospital, New Taipei City 23148, Taiwan; m212092001@tmu.edu.tw; 5 School of Medicine, Fu Jen Catholic University, New Taipei City 242062, Taiwan; 6 Department of Neurosurgery, National Taiwan University, Yunlin Branch, Yunlin 640203, Taiwan

**Keywords:** CASK, hydrogen peroxide, parthanatos, mitochondrial dysfunction, EGFR, microglia, PARP-1, AKT, AMPK

## Abstract

Calcium/calmodulin-dependent serine protein kinase (CASK) is a scaffold protein and plays critical roles in neuronal synaptic formation and brain development. Previously, CASK was shown to associate with EGFR to maintain the vulval cell differentiation in *C. elegans*. In this study, we explored the role of CASK in CHME3 microglial cells. We found that CASK silencing protects cells from H_2_O_2_-induced cell death by attenuating PARP-1 activation, mitochondrial membrane potential loss, reactive oxygen species production, and mitochondrial fission, but it increases oxidative phosphorylation. The PARP-1 inhibitor olaparib blocks H_2_O_2_-induced cell death, suggesting the death mode of parthanatos. CASK silencing also increases AKT activation but decreases AMPK activation under H_2_O_2_ treatment. Pharmacological data further indicate that both signaling changes contribute to cell protection. Different from the canonical parthanatos pathway, we did not observe the AIF translocation from mitochondria into the nucleus, suggesting a non-canonical AIF-independent parthanatos in H_2_O_2_-treated CHME3 cells. Moreover, we found that CASK silencing upregulates the EGFR gene and protein expression and increases H_2_O_2_-induced EGFR phosphorylation in CHME3 microglia. However, EGFR activation does not contribute to cell protection caused by CASK silencing. In conclusion, CASK plays a crucial role in microglial parthanatos upon H_2_O_2_ treatment via stimulation of PARP-1 and AMPK but the inhibition of AKT. These findings suggest that CASK might be an ideal therapeutic target for CNS disorders.

## 1. Introduction

Calcium/calmodulin-dependent serine protein kinase (CASK) is a multidomain scaffold protein, which belongs to the membrane-associated guanylate-kinase protein (MAGUK) family [1]. CASK was initially identified from neuronal cells. The human RNA Seq database reveals that the mRNA and protein expression of CASK are ubiquitously detectable in different regions of brain tissue [2]. The human CASK gene is located on the short arm of the X chromosome and numerous studies indicate that the mutant CASK gene leads to the progression of multiple neuronal disorders, including X-linked intellectual disability, microcephaly with pontine and cerebellar hypoplasia, and developmental delay [3]. In neuronal cells, CASK is localized in the nucleus and enriched at the plasma membrane of the pre- and postsynapses. CASK performs its functions by association with a wide range of interaction partners [4,5]. Moreover, the Kyoto Encyclopedia of Genes and Genomes (KEGG) analysis illustrates that the protein changes in the whole brain of CASK-mutant mice are highly associated with the neurodegenerative disorder [6]. 

The beneficial role of CASK on synapses has been extensively demonstrated. At the presynaptic sites, CASK is responsible for the regulation of synaptic vesicle exocytosis and neurotransmitter release through the interaction between the CASK-neurexin-1β-Mint1 tripartite complex and liprin-α [7,8]. CASK also contributes to maintaining the structure of postsynaptic dendritic spines by serving as a scaffold protein connecting the syndecan-2 with the F-actin cytoskeleton [9]. Other than the scaffolding role of CASK in the neurons, CASK was recently reported to promote neuronal cell apoptosis through phosphorylating α-synuclein and activating the mitochondrial calcium uniporter, thereby leading to mitochondrial damage and cell death [10]. Besides the crucial functions in the neurons, CASK regulates a diverse range of functions in different tissues. A few studies have highlighted the interaction between CASK and epidermal growth factor receptor (EGFR). In *C. elegans*, CASK was found to be essential in the process of vulval development. CASK promotes the proliferation and differentiation of vulval precursor cells [11] and stabilizes the basolateral localization of EGFR in vulval cells [12], both of which contribute to vulval organogenesis [13]. 

Microglia serve as immune sentinels in the CNS, not only surveilling and scavenging pathogens but also regulating neuronal activity through pruning the synapses of neuronal circuits. Upon activation by the aberrant pathological stimulus, the microglia start to induce the immune response and extensively generate inflammatory mediators that contribute to neuroinflammation and neurotoxicity [14]. Numerous studies have demonstrated that the elevated reactive oxygen species (ROS) production during oxidative stress plays a critical role in the activation of microglial cells which subsequently generate more ROS and amplify the oxidative damage [15,16,17]. The ROS overproduction and neuroinflammation in microglia are frequently associated with neuronal disorders [14,18,19]. Besides the microglia-derived ROS generation and inflammatory response that play an indispensable role in the progression of neuronal disorders, the occurrence of microglial cell death also fosters the development of neurodegeneration. Studies indicate that under various stressful conditions, the microglia are more vulnerable to death, compared to the peripheral macrophages [20]. Additionally, the apoptosis of microglial cells accelerates neuronal loss, β-amyloid accumulation, and the development of Alzheimer’s disease [21]. Moreover, the PARP-1 overactivation in the microglia induced by oxygen-glucose deprivation, glutamate, and DNA alkylating agents leads to ROS synthesis and parthanatos [22,23]. 

EGFR is widely expressed in the mammalian CNS including neurons and glial cells, and it regulates microglia-mediated inflammation in the brain [24]. Upon LPS treatment and spinal cord injury, primary microglia and BV-2 murine cells show enhanced EGFR and downstream MAPK activation, accompanied by the generation of inflammatory mediators. The inhibition of EGFR and MAPK phosphorylation significantly attenuates LPS- and spinal cord injury-induced proinflammatory cytokine generation, leading to morphological and neurological recovery [25]. On the other hand, secreted phospholipase A2-IIA, a protein playing an important role in the development of Alzheimer’s disease, was found to be induced by EGFR in microglial cells and involved in cell proliferation, phagocytosis, and inflammatory response [26]. In addition, EGFR tyrosine kinase inhibitor AG1478 was found to abrogate morphine-induced microglial EGFR activation, cell reactivity, migration, and inflammatory response [27]. Likewise, LPS-mediated microglial cell migration is dependent on the EGFR activation [28].

To date, the cellular function of CASK in microglia has not been investigated yet. Because CASK mediates EGFR action in vulval development in *C. elegans* [11,12,13], we were interested to understand the cellular functions of CASK in microglia under oxidative stress and to decipher its possible relationship with EGFR. The cell model we used in this study is human immortalized microglial cell line clone 3 (CHME3). 

## 2. Materials and Methods

### 2.1. Reagents and Antibodies

Dulbecco’s Modified Eagle Medium, trypsin/EDTA solution, and fetal bovine serum (FBS) were all purchased from Gibco (Carlsbad, CA, USA). Penicillin-streptomycin antibiotic solution was bought from Biological Industries (Kibbutz Beit-Haemek, Israel). H_2_O_2_ solution was ordered from Honeywell (Morris Plains, NJ, USA). Dulbecco’s phosphate-buffered saline (PBS), sodium bicarbonate, puromycin, protease inhibitor cocktails, oligomycin, FCCP, rotenone, MG132, zVAD-FMK (carbobenzoxy-valyl-alanyl-aspartyl-[O-methyl]-fluoromethylketone), and 3MA (3-methyladenine) were obtained from Sigma-Aldrich (St. Louis, MO, USA). Necrostatin-1 (Nec-1), olaparib, and gefitinib were ordered from Selleckchem (Houston, TX, USA). Cycloheximide (CHX), U0126, and bafilomycin A1 (BafA1) were bought from Calbiochem (San Diego, CA, USA). A769622 was purchased from Tocris (Bristol, UK). SP600125, SB203580, and AKT-IN-VIII were bought from MedChemExpress. Antibodies of p-EGFR (Y1068) (#3777S), γH2AX (#9718), PARP-1 (#9542), TOM20 (#42406), Mitotracker (#9082P), p-JNK (Thr183/Tyr185) (#9251), p-ERK (Thr202/Tyr204) (#9101), p-p38 (Thr180/Tyr182) (#9211), p-AKT (Ser473) (#4060S), AKT (#9272S), and p-AMPK (Thr172) (#2531S) were purchased from Cell Signaling Technology (Beverly, MA, USA). Antibodies of CASK (sc-13158), β-actin (sc-47778), JNK (sc-1648), ERK (sc-154), p38 (sc-81621), AMPK (sc-74461), and AIF (sc-13116) were obtained from Santa Cruz Biotechnology (Dallas, TX, USA). The antibody of PAR (4335-MC-100) was purchased from R&D Systems (Minneapolis, MN, USA). The antibody of EGFR (06-847) was bought from Merck Millipore (Burlington, MA, USA). The antibody of LC3 (GTX127375) was purchased from GeneTex (Irvine, CA, USA). 

### 2.2. Cell Culture

The human CHME3 microglial cell line was purchased from ATCC (CRL-3304) and cultured in high-glucose DMEM supplemented with 10% of FBS and 1% of penicillin-streptomycin antibiotics solution. Cells were incubated in a humidified incubator at 37 °C and 5% of CO_2_ in air. 

### 2.3. Generation of Short Hairpin RNA (shRNA) Knockdown Cell Line

CHME3 microglia were transfected with the lentivirus encoding shRNA targeting CASK (CCCTGAGAATAACGACGCAAA) and EGFR (GCTGGATGATAGACGCAGATA) in the DMEM containing 10% of FBS and 8 μg/mL polybrene but without the addition of penicillin-streptomycin solution. After 24 h infection, the medium was removed and replaced with a fresh complete DMEM that contained 3 μg/mL puromycin for cell selection purposes. The medium was replenished every 3 days and the selection process lasted for 1 week. The knockdown of CASK and EGFR was determined using Western blot. The CASK and EGFR knockdown efficiencies of 70% and 80% were quantified using Imagelab 6.1 analysis software. Lastly, the stable CASK or EGFR silencing CHME3 cell lines were maintained with puromycin (1 μg/mL) for every future subculture. 

### 2.4. Flow Cytometry for Annexin V/PI Staining

CHME3 cells were seeded at 1 × 10^5^ per well in 6-well plates and incubated in complete medium overnight. After drug treatment, the medium was collected and the attached cells were trypsinized and collected into eppendorf. The medium containing the cells was centrifuged at 1500 rpm for 6 min. The supernatant was removed after centrifuging and the cells were stained with 100 μL Annexin V binding buffer containing 5 μL of Annexin V and 10 μL of propidium iodide (PI) for 20 min at room temperature. After finishing the staining process, the cell samples were transferred from the Eppendorf tubes to flow tubes, and 400 μL of Annexin V binding buffer was added. The cell viability was determined with a flow cytometer (BD FACSCalibur, Franklin Lakes, NJ, USA) with the FL1 and FL2 bivariate analyses. The results were analyzed using the CellQuest Pro software 4.0.1 by calculating the percentage of cells in the lower left quadrants as living cells. 

### 2.5. Intracellular ROS Production and Mitochondrial Membrane Potential Measurements

The H_2_O_2_-induced ROS production was measured with DHE (5 μM) and MitoSOX (5 μM), which indicate the expression of cytosol- and mitochondrial-derived ROS, respectively. The mitochondrial membrane potential (MMP) was determined with JC-1 staining. Cells were seeded in 6-well plates and incubated overnight in 1 mL of complete DMEM medium per well. After finishing the treatment, the medium was collected and the attached cells were trypsinized and collected into Eppendorf tubes. The medium containing the cells was centrifuged at 1500 rpm for 6 min. The supernatant was removed after centrifuging and followed by staining at 37 °C for 30 min. The cells were next sent to the centrifuge at 1500 rpm for 6 min and the supernatant was removed to collect the cell pellet. Lastly, the cell pellet was resuspended with 500 μL PBS and transferred to the flow tube. The sample for ROS production was measured with a flow cytometer (BD FACSCalibur, Franklin Lakes, NJ, USA) with the FL2 analysis, while the MMP was measured with the FL1 and FL2 bivariate analyses. 

### 2.6. Immunoblotting

After finishing the drug treatment, the medium was removed and the cells were washed with PBS. The cells were next lysed into the RIPA lysis buffer that consisted of 50 mM Tris-HCL pH 7.6, 150 mM NaCl, 1% Triton X-100, 0.1% sodium dodecylsulfate (SDS), 0.1% deoxycholate, 2 mM NaF, 2 mM Na_3_VO_4_, 1 mM phenylmethylsulfonyl fluoride (PMSF), and 0.2% PI cocktails. After lysing, the cells were sonicated with 6 interval pulses and centrifuged at 13,000 rpm at 4 °C for 15 min. The supernatant was collected to quantify the protein concentration using the Bio-Rad protein assay and each sample was normalized into equal concentration with the addition of RIPA buffer. Lastly, the samples were mixed with 1X sample loading buffer and heated at 98 °C for 8 min. 

Approximately 20 μg protein was loaded and separated according to its respective molecular weight in the 8–15% SDS-PAGE gel. The gel containing separated protein was transferred onto Immobilon-P transfer membrane (Millipore, Bedford, MA, USA) and next blocked and shaken gently with 5% non-fat milk in Tris-buffer saline (TBS) with 0.1% Tween 20 for 1 h at room temperature. The membrane was incubated with primary antibodies and shaken gently overnight at 4 °C. The primary antibodies were removed from the membrane after overnight shaking and the membrane was washed three times with an interval time of 10 min using TBST. After washing, the membrane was incubated with horseradish-peroxidase-linked secondary antibodies with gentle shaking for 1 h at room temperature. The membrane was washed with TBST again three times, and lastly, the protein bands on the membrane were detected on the Bio-RAD UVP system with the enhanced chemiluminescence reagents. Western blotting images were quantified using Imagelab analysis software and β-actin was used as the internal control for every protein quantification. 

### 2.7. Reverse Transcription (RT) and Real-Time Polymerase Chain Reaction (RT-PCR)

After drug treatment, the cells were washed with PBS and lysed with 300 μL Trizol reagent (Invitrogen, Shanghai, China). The Trizol sample was added with 60 μL chloroform and vortexed for 30–60 s before being sent to centrifuge at 12,500 rpm for 15 min. An amount of 150 μL extracted supernatant was collected and 150 μL isopropanol was mixed well and stored at 20 °C overnight. The sample on the other day was first centrifuged, followed by the washing process with 300 μL 75% EtOH in DEPC water. After washing, the supernatant was removed and the RNA pellet was air-dried at room temperature for 20 min. An amount of 2 μg total RNA underwent the RT process and was converted into cDNA. The converted cDNA was mixed with SYBR Green Master Mix, targeted gene primers (Table 1), and ddH_2_O to apply RT-PCR through operating the QuantStudio 5 system (Applied Biosystems, Oakland, CA, USA). The primers used to amplify the targeted genes are demonstrated below. Β-Actin was used to normalize the mRNA fold changes of the targeted genes. 

### 2.8. Confocal Microscopy

Sterilized glass slides were placed into the 12-well plate and sterilized again under UVB light for 30 min before seeding the cells. After drug treatment, the cells were washed with PBS, fixed with 4% paraformaldehyde for 15 min, and permeabilized with 0.2% Triton X-100 in PBS for 20 min. The samples were incubated with 4% BSA to block nonspecific binding and incubated with primary antibodies at 4 °C overnight. The primary antibodies were removed and the cells were washed with PBS 3 times. After washing, the cells were immunostained with the fluorochrome-conjugated secondary antibody for 1 h under the dark environment. Lastly, the coverslips were counterstained with 4′,6-diamidino-2-phenylindole (DAPI) and mounted on the microscope slides. The prepared samples were analyzed by a LSM780 confocal microscope (Zeiss, Shanghai, China). To determine mitochondrial network, we used Imaris 9.8 (Oxford) to calculate the volume of different types of mitochondria.

### 2.9. Mitochondrial Oxygen Consumption Rate (OCR)

The cells were seeded in the Seahorse 24-well V7 microplate and cultured in complete DMEM overnight. After drug treatment, the medium was removed and the cells were incubated in XF assay medium in the absence of NaHCO_3_ at 37 °C for 1 h in the measuring chamber without CO_2_ input. The mitochondrial complex inhibitors [oligomycin (2.5 μM), rotenone (2.5 μM), antimycin A1 (2.5 μM), and FCCP (1 μM)] were freshly prepared in XF assay media. OCR was recorded as pMoles per minute. The ATP turnover was examined after the oligomycin treatment. Respiratory capacity was the maximum rate after FCCP treatment subtracted by the non-mitochondrial respiration. All the measurements and calculations were analyzed with Seahorse XFe24 wave software version Wave 2.2 (Seahorse Bioscience) according to the protocol from the manufacturer. 

### 2.10. Statistical Analysis

Data were all presented as the mean ± S.E.M of independent experiments. Prism 9 software was utilized for the statistical analysis. Multiple *t*-tests were selected to assess the significance of the differences between samples, and *p* < 0.05 was considered to be statistically significant. Besides using multiple *t*-tests to assess the significant difference between shCTL and shCASK cells under different treatment conditions, we also utilized one-way ANOVA to analyze the data.

## 3. Results

### 3.1. CASK Silencing Attenuates H_2_O_2_-Induced Cell Death in CHME3

**Figure 1:**
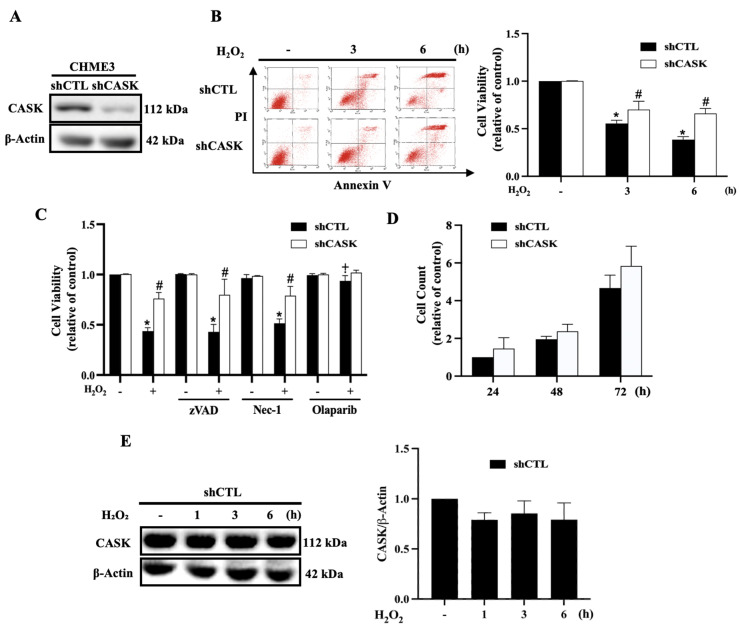
CASK silencing attenuates H_2_O_2_-induced cell death in CHME3 microglia. (**A**) CASK protein levels in CHME3 in shCTL and shCASK conditions were determined by immunoblotting. (**B**) CHME3 cells were treated with H_2_O_2_ (500 μM) for 3 and 6 h, then cell viability was determined by Annexin V/PI staining. (**C**) CHME3 cells were pretreated with zVAD (20 μM), necrostatin-1 (10 μM), or olaparib (10 μM) for 30 min followed by treatment with H_2_O_2_ (500 μM) for 6 h. Cell viability was determined by Annexin V/PI staining. (**D**) CHME3 cells were cultured in complete medium for indicated times, and viable cells were counted by trypan blue staining. (**E**) After H_2_O_2_ (500 μM) treatment for indicated times, CASK protein levels were determined by immunoblotting. The data were mean ± S.E.M. from at least 3 independent experiments. *, *p* < 0.05 indicates a significant effect of H_2_O_2_ compared to the non-treated group. #, *p* < 0.05 indicates a significant effect of CASK silencing compared to shCTL. ⨥, *p* < 0.05 indicates a significant effect of olaparib to protect cell death caused by H_2_O_2_ in shCTL cells.

In order to study the role of CASK in microglia under oxidative stress, we utilized the human CHME3 cell line for our experiments. We initially confirmed the expression of CASK in the CHME3 cell line and subsequently generated a CASK silencing cell line through lentivirus transfection in CHME3 cells (Figure 1A). In our study, H_2_O_2_ was selected as an oxidative stress inducer. We first observed that H_2_O_2_ (500 μM) treatment for 3 h was capable of inducing cell death in CHME3 microglia cells. However, the death effect of H_2_O_2_ treatment up to 6 h was significantly attenuated by shCASK (Figure 1B). Next, we found that the PARP-1 inhibitor olaparib (10 μM) significantly reversed the cell death in both shCTL and shCASK cells. In contrast, zVAD (20 μM) and necrostatin-1 (10 μM) had no effects on H_2_O_2_-induced cell death (Figure 1C). This suggests that the H_2_O_2_-induced cell death in CHME3 is parthanatos, which is characterized by PARP-1 hyperactivation. Additionally, we investigated whether CASK is involved in the regulation of microglial cell growth, thereby affecting the cell susceptibility to H_2_O_2_ insult. In this aspect, we observed that CASK silencing did not alter the cell growth within the culture for up to 72 h (Figure 1D). Furthermore, our data revealed that the CASK protein expression was not significantly changed under the H_2_O_2_ treatment in CHME3 cells (Figure 1E). 

### 3.2. CASK Silencing Reduces H_2_O_2_-Induced DNA Damage and PAR Formation in CHME3 Microglia

**Figure 2:**
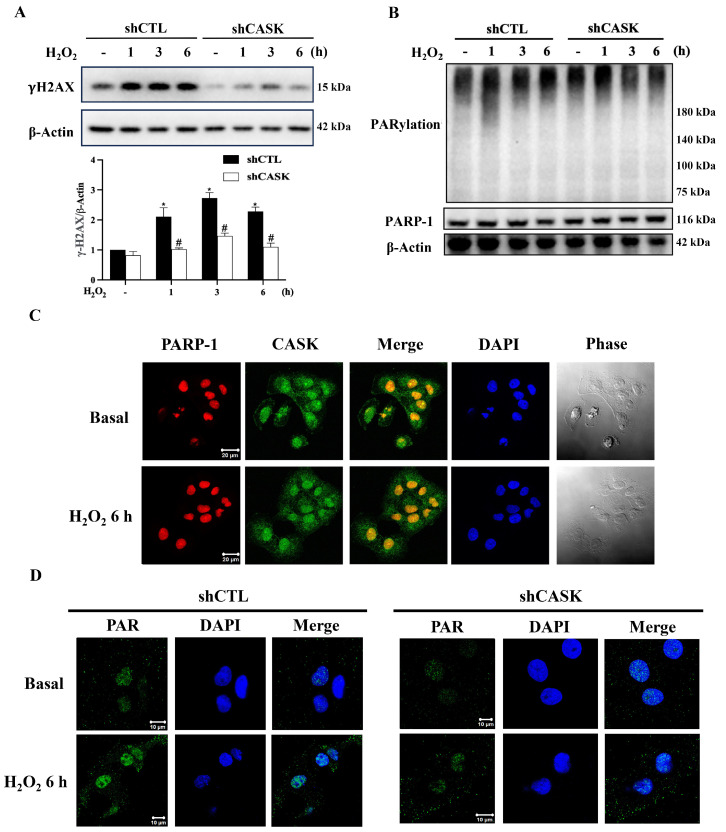
H_2_O_2_-induced DNA damage and PARP-1 activation are attenuated by shCASK in CHME3. The cells were treated with H_2_O_2_ (500 μM) for the indicated times. (**A**,**B**) Immunoblotting of γH_2_AX (**A**) and PAR (**B**) was conducted. (**C**,**D**) Confocal microscopic examinations of PARP-1, CASK (**C**), and PAR (**D**) were taken. The data were mean ± S.E.M. from at least 3 independent experiments. *, *p* < 0.05 indicates a significant effect of H_2_O_2_ compared to the non-treated group. #, *p* < 0.05 indicates a significant effect of shCASK compared to shCTL. The scale bars are 20 μm in (**C**) and 10 μm in (**D**).

PARP-1 overactivation is the initial and the most decisive step in the parthanatic cascade [29]. Toxic stimuli, such as H_2_O_2_ and hydroxyl radicals, are capable of inducing nuclear DNA damage and causing PARP-1 overactivation [30]. In our study, we found that CASK silencing attenuated H_2_O_2_-induced CHME3 parthanatos. To confirm this notion, we assessed the ability of H_2_O_2_ to induce γH2AX expression, an index of DNA damage within 6 h of treatment, and found that this effect was significantly reduced in shCASK cells (Figure 2A). Moreover, PARylation, a result of PARP-1 activation, was induced at 1 h and gradually returned to the baseline level at 6 h after H_2_O_2_ treatment. This effect was also attenuated by CASK silencing under the conditions where the PARP-1 protein expression was not altered by H_2_O_2_ treatment (Figure 2B).

Moreover, the confocal microscopic results showed that PARP-1 as expected was majorly present in the nucleus. However, CASK was localized in the nuclei and cytosol before and after H_2_O_2_ treatment (Figure 2C). Moreover, H_2_O_2_ can induce significant PAR accumulation in the nucleus and moderate PAR appearance in the cytosol, while CASK silencing impaired such PAR formation effect (Figure 2D). Conclusively, CASK exerts a positive role to regulate DNA damage and PARP-1 activation induced by H_2_O_2_. 

### 3.3. CASK Silencing Inhibits H_2_O_2_-Induced Mitochondrial Dysfunction and Generation of Mitochondrial and Cytosolic ROS

**Figure 3:**
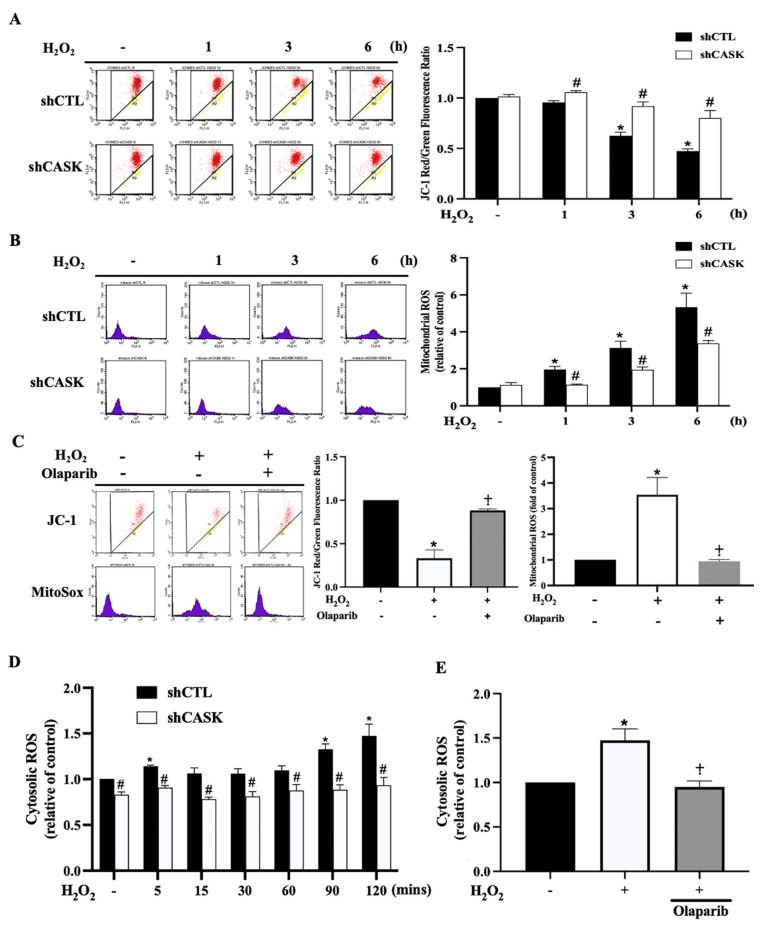
CASK silencing attenuates H_2_O_2_-induced PARP-1-dependent MMP loss and ROS production. (**A**,**B**,**D**) Control and shCASK cells were treated with H_2_O_2_ (500 μM) for indicated times, and MMP (**A**), mitochondrial ROS production (**B**), and cytosolic ROS level (**D**) were determined by JC-1, MitoSOX, and DHE, respectively. (**C**,**E**) Cells were pretreated with olaparib (10 μM) for 30 min, followed by H_2_O_2_ (500 μM) for 6 h (**C**) and 120 min (**E**). MMP (**A**,**C**), mitochondrial ROS level (**B**,**C**), and cytosolic ROS level (**D**,**E**) were determined. The data were mean ± S.E.M. from at least 3 independent experiments. *, *p* < 0.05 indicates a significant effect of H_2_O_2_ compared to the non-treated group. #, *p* < 0.05 indicates a significant effect of shCASK compared to shCTL. ⨥, *p* < 0.05 indicates a significant effect of olaparib compared to H_2_O_2_ response in shCTL cells.

Mitochondria are an important organelle in the regulation of parthanatos-associated events. Excessive PAR generation triggers the loss of mitochondrial integrity and facilitates the release of AIF from mitochondria to the nucleus to induce parthanatos. Hence, we further explored whether CASK participates in the regulation of mitochondrial functionality and ROS generation. Firstly, we found that MMP was significantly lost under the H_2_O_2_ treatment, and CASK silencing prevented the MMP loss (Figure 3A). Additionally, the time-dependent increase in mitochondrial ROS (mtROS) generation induced by H_2_O_2_ was reduced by CASK silencing (Figure 3B). Moreover, our data revealed that the H_2_O_2_-induced effects were abrogated by PARP-1 inhibitor olaparib (Figure 3C). Meanwhile, the cytosolic ROS production induced by H_2_O_2_ was also markedly inhibited by CASK silencing (Figure 3D) and olaparib (Figure 3E). These results indicate that the cell protective effect of CASK silencing might be derived from the inhibition of PARP-1-dependent mitochondrial dysfunction. 

### 3.4. CASK Silencing Attenuates OXPHOS Loss and Mitochondrial Fission under H_2_O_2_ Treatment

**Figure 4:**
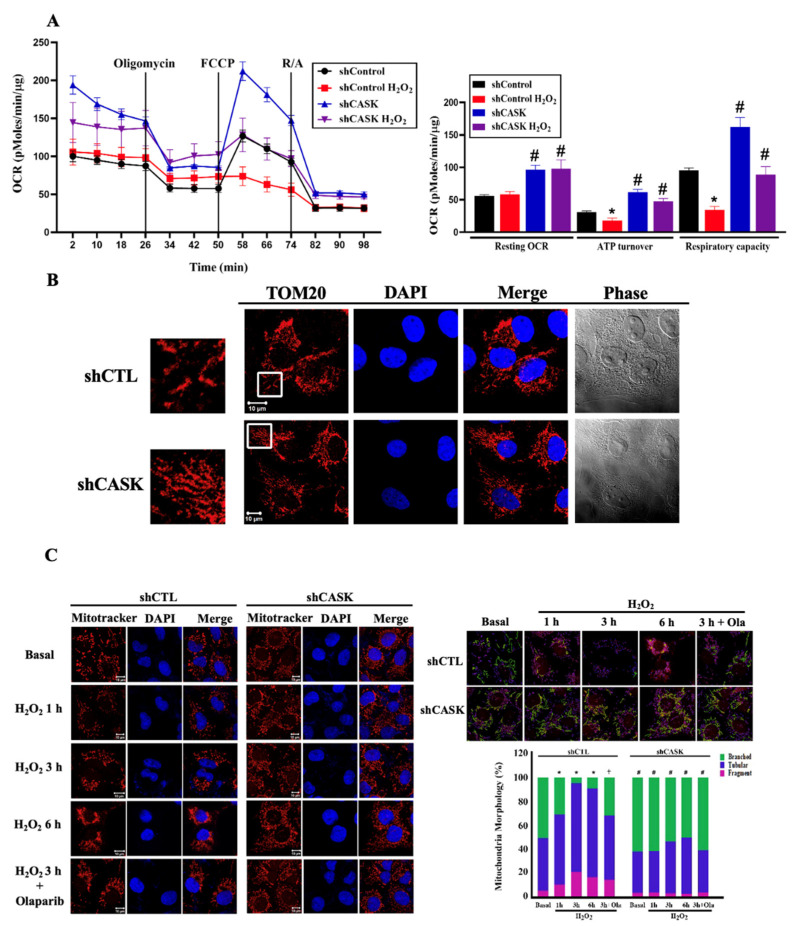
CASK silencing attenuates H_2_O_2_-induced OXPHOS loss and mitochondrial fission. (**A**) After treatment with H_2_O_2_ (500 μM) for 1 h, CHME3 cells were subjected to the Seahorse apparatus for measuring OCR. (**B**) Mitochondrial morphology in CHME3 cells was determined by staining with TOM20 and examined by confocal microscopy. (**C**) CHME3 cells were pretreated with vehicle or olaparib (10 μM) followed by H_2_O_2_ (500 μM) for the indicated times. Mitochondrial morphology was determined by staining with Mitotracker and examined by confocal microscopy. The scale bar is 10 μm in (**B**,**C**). The percentage of the branched mitochondria shown in the green bar was statistically analyzed. *, *p* < 0.05 indicates a significant effect of H_2_O_2_ compared to the non-treated group. #, *p* < 0.05 indicates a significant effect of shCASK compared to shCTL. ⨥, *p* < 0.05 indicates a significant effect of olaparib in shCTL cells.

Since CASK silencing in CHME3 microglia significantly maintains the mitochondrial function under H_2_O_2_ treatment, we next would like to uncover whether CASK silencing also regulates mitochondrial respiration. The Seahorse assay was carried out to determine the mitochondrial OCR before and after H_2_O_2_ treatment. Our results revealed that CASK silencing significantly improved basal OCR, ATP turnover, and respiratory capacity. Additionally, the ATP turnover and respiratory capacity that were markedly reduced under H_2_O_2_ treatment for 1 h were blocked by CASK silencing (Figure 4A). This implies that CASK plays a crucial role in regulating mitochondrial respiration. 

Previous study demonstrated that the H_2_O_2_-induced parthanatos is associated with increased mitochondrial fission in ARPE-19 cells [31]. Therefore, we also examined whether CASK is involved in the regulation of mitochondrial dynamics. In the resting state condition, we observed that CASK silencing markedly enhanced the mitochondrial integrity under H_2_O_2_ treatment (Figure 4B). Additionally, H_2_O_2_ treatment induced mitochondrial fission in a time-dependent manner, while CASK silencing and olaparib significantly inhibited this effect of H_2_O_2_ (Figure 4C). The mitochondrial morphology was divided into branched, tubular, and fragment three categories and quantified using Imaris 9.8 (Oxford). (Figure 4C). The branched mitochondria indicating the fusion was decreased time-dependently by H_2_O_2_ and this effect was reversed by olaparib and CASK silencing. 

### 3.5. AIF Does Not Translocate into the Nucleus after H_2_O_2_ Treatment

**Figure 5:**
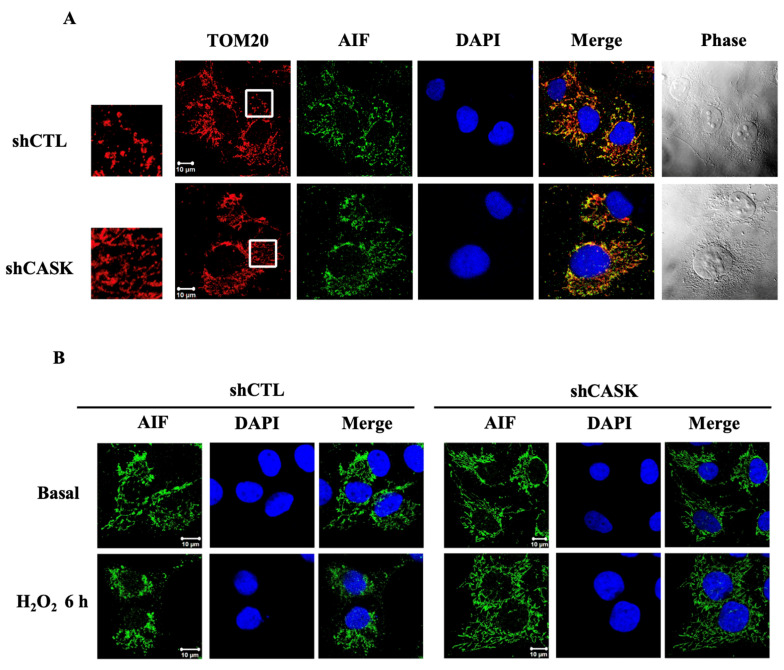
H_2_O_2_ does not induce nuclear translocation of AIF. The subcellular localization of AIF before and after H_2_O_2_ (500 μM) treatment was determined. (**A**) The localization of AIF in mitochondria was examined. (**B**) AIF remained in the mitochondria, not entering nuclei, after H_2_O_2_ treatment. The scale bar is 10 μm.

In the classical parthanatos procedure, the PARP-1-mediated AIF release from mitochondria forms a dimer with MIF and the dimers translocate into the nucleus and cleave DNA into fragmentation. Here, we determined whether CASK might affect the translocation of AIF under H_2_O_2_ treatment. As expected, AIF was localized in the mitochondria and CASK silencing did not alter the expression of AIF at the resting state (Figure 5A). Moreover, after H_2_O_2_ treatment for 6 h, AIF was still present in the mitochondria and was not seen in the nucleus (Figure 5B). These findings suggest that unlike classical parthanatos, H_2_O_2_ does not induce AIF translocation from mitochondria to the cytosol and the nucleus. 

### 3.6. Autophagy Is Not Involved in H_2_O_2_-Induced Microglial Cell Death

Because H_2_O_2_ induces mitochondrial dysfunction, we wondered if mitophagy occurs. Therefore, we first measured LC3, whose conversion from LC3-I to LC3-II is regarded as an index of autophagic flux. Our results revealed that the LC3-II/I ratio was not significantly altered under H_2_O_2_ treatment (Supplementary Figure S1A). In line with this finding, we did not detect any effect of H_2_O_2_ on lysosomal mass (Supplementary Figure S1B). Moreover, two autophagy inhibitors, BafA1 and 3-MA, did not change H_2_O_2_-induced cell death in shCTL and shCASK cells (Supplementary Figure S1C).

### 3.7. AMPK Inhibition and AKT Activation Contribute to the Cell Protection Effect of CASK Silencing

**Figure 6:**
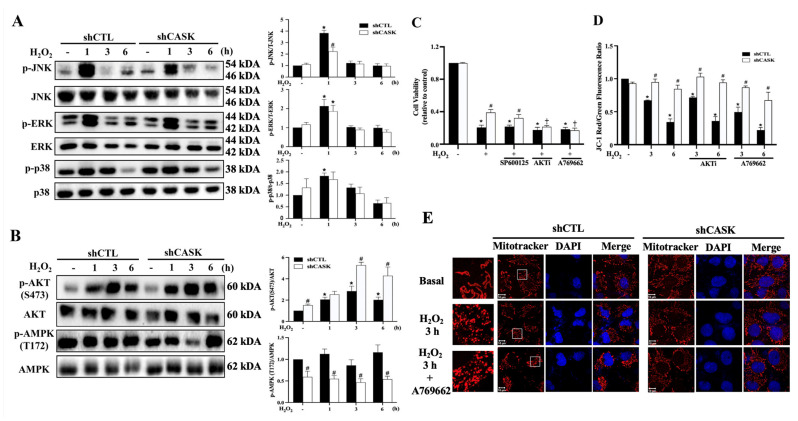
CASK silencing affects H_2_O_2_-induced signaling pathways, and it increased AKT activation but decreased AMPK activation to contribute to the cell protective effect of CASK silencing. (**A**,**B**) CHME3 cells were treated with H_2_O_2_ (500 μM) for the indicated times. Immunoblotting was conducted to measure JNK, ERK, and p38 (**A**) and AKT and AMPK (**B**), and then quantification analysis was taken from 3 independent experiments. (**C**) CHME3 cells were pretreated with SP600125 (10 μM), AKT-IN-VIII (AKTi, 10 μM), or A769622 (25 μM) for 20 min followed by stimulation with H_2_O_2_ (500 μM) for 6 h. Cell viability was determined by Annexin V/PI staining. (**D**) CHME3 cells were pretreated with AKTi (10 μM) or A769622 (25 μM) for 30 min followed by stimulation with H_2_O_2_ (500 μM) for the indicated times. MMP was determined by JC-1 staining and flow cytometry. (**E**) CHME3 cells were pretreated with A769622 (25 μM) for 30 min followed by the stimulation with H_2_O_2_ (500 μM) for 3 h. Mitochondrial morphology was measured by staining with Mitotracker in confocal microscopy. The data were mean ± S.E.M. from at least 3 independent experiments. *, *p* < 0.05 indicates a significant effect of H_2_O_2_ compared to the non-treated group. #, *p* < 0.05 indicates the significant effects of shCASK compared to shCTL. ⨥, *p* < 0.05 indicates the significant effects of AKTi and A769662 to block the cell protective effect of CASK silencing. The scale bar is 10 μm in (**E**).

Multiple studies demonstrate that the MAPK pathways including JNK, ERK, and p38 are associated with the parthanatos cell death cascades [32,33,34]. Furthermore, AKT and AMPK are also reported to participate in the progression of parthanatos cell death [35,36]. Here, we would like to understand the signaling pathway that is regulated by CASK and involved in the H_2_O_2_-induced parthanatic cell death. Under H_2_O_2_ treatment, JNK, ERK, and p38 were transiently activated at 1 h and we found that CASK silencing only attenuated JNK phosphorylation (Figure 6A). Furthermore, H_2_O_2_ treatment also induced AKT phosphorylation but barely affected AMPK activation. We found that CASK silencing enhanced H_2_O_2_-induced AKT phosphorylation, while it inhibited AMPK activation (Figure 6B). 

After observing the effects on JNK, AKT, and AMPK signaling, we would like to understand how these signaling pathways regulate cell death under H_2_O_2_ treatment. We found that AKT inhibitor (10 μM AKT-IN-VIII) can reduce the protective effect of CASK silencing, while JNK inhibitor (10 μM SP600125) cannot. In addition, AMPK activator A769662 (25 μM) abolished the protective effect of CASK silencing (Figure 6C). Nevertheless, we found that A769662 and AKT-IN-VIII failed to change H_2_O_2_-induced MMP loss in shCTL cells and CASK silencing-induced MMP protection (Figure 6D). Moreover, we found that A769662 did not alter the mitochondrial fission induced by H_2_O_2_ in shCTL cells, nor the CASK silencing-induced mitochondrial fusion (Figure 6E). Collectively, we suggest AKT activation and AMPK inhibition involve in the cell protective effect of CASK silencing. Nevertheless, the maintenance of mitochondrial dynamics and MMP by CASK silencing are independent of AMPK and AKT activation. 

### 3.8. CASK Silencing Enhances EGFR Expression While EGFR Activation Is Not Involved in the Action of CASK Silencing

**Figure 7:**
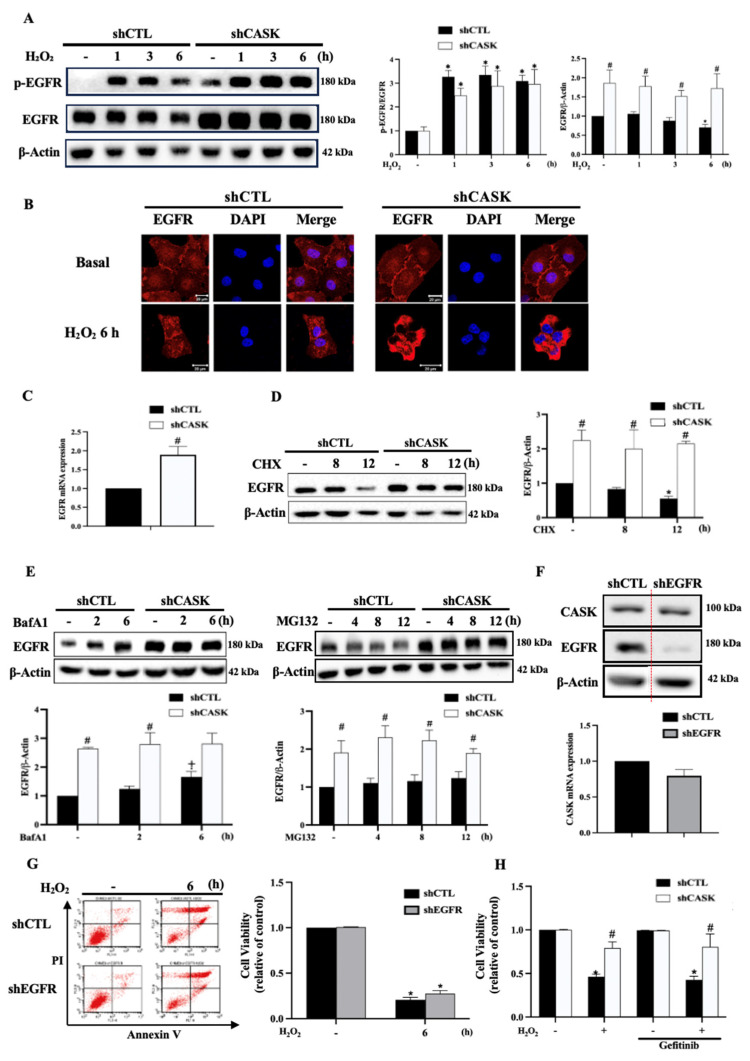
CASK silencing upregulates EGFR expression, but EGFR is not involved in cell death control. (**A**) CHME3 cells were stimulated with H_2_O_2_ (500 μM) for the indicated times. Immunoblotting was conducted to determine EGFR. EGFR and p-EGFR/EGFR levels were quantified. (**B**) After H_2_O_2_ (500 μM) treatment for 6 h, the immunostaining of EGFR and DAPI was conducted and detected by confocal microscopy. (**C**) EGFR gene expression in control and CASK silencing cells was determined by real-time PCR. (**D**,**E**) After treatment with cycloheximide (CHX, 10 μg/mL) (**D**), bafilomycin A1 (100 nM), or MG132 (10 μM) (**E**) for different times, EGFR level was determined by immunoblotting. (**F**) CASK protein and mRNA levels were measured in control and EGFR silencing cells. (**G**) Control and EGFR silencing cells were treated with H_2_O_2_ (500 μM) for 6 h. (**H**) Control and CASK silencing cells were pretreated with gefitinib (5 μM) for 30 min followed by H_2_O_2_ (500 μM) treatment for 6 h. Cell viability was determined by Annexin V/PI staining. The data were mean ± S.E.M. from at least 3 independent experiments. *, *p* < 0.05 indicates a significant effect of H_2_O_2_ compared to the non-treated group. #, *p* < 0.05 indicates a significant effect of CASK silencing compared to shCTL. ⨥, *p* < 0.05 indicates a significant effect of BafA1 to increase EGFR expression in shCTL cells. The scale bar is 20 μm in (**B**).

EGFR phosphorylation is associated with the development of neuronal disorders. Multiple studies show the beneficial recovery outcome upon the inhibition of EGFR phosphorylation in microglia [24,25,28,37]. A previous study in Drosophila showed that CASK homolog LIN-2 is involved in targeting the localization of EGFR homolog LET-23 on the basolateral membrane, suggesting the role of CASK in regulating EGFR activity [12]. This urges us to further determine the relationship between CASK and EGFR in microglia. First, we found that H_2_O_2_ time-dependently induced EGFR transactivation was accompanied by a late and slight downregulation of EGFR after H_2_O_2_ treatment for 6 h. Intriguingly, CASK silencing enhanced the EGFR protein level at the basal condition and this effect was maintained after H_2_O_2_ treatment (Figure 7A). Confocal microscopic data further showed that EGFR was present in the nuclei, cytosol, and plasma membrane at the resting state. After H_2_O_2_ treatment, EGFR moved to the cytosol. Moreover, the EGFR immunoactivity in shCTL cells was weaker than that in shCASK cells (Figure 7B). These findings suggest that H_2_O_2_ can activate EGFR in CHME3 cells, and CASK silencing can enhance this effect. 

After observing the effect of CASK silencing to upregulate EGFR, we determined EGFR gene expression. We observed that the EGFR mRNA level was increased in the CASK silencing CHME3 microglia (Figure 7C). We also found that the EGFR protein stability in CHME3 cells was enhanced by the CASK silencing. Under cycloheximide treatment, the EGFR level in shCTL cells was downregulated to around 50% at 12 h, while this effect was not seen in shCASK cells (Figure 7D). Further using inhibitors of lysosomes and proteasomes, i.e., bafilomycin A1 and MG132, respectively, we found that only bafilomycin A1 increased the EGFR protein level in shCTL cells, and this increasing effect was no longer detected in shCASK cells (Figure 7E). Therefore, we suggest that CASK silencing increases EGFR expression via at least two mechanisms, including the induction of EGFR gene transcription and the decrease in EGFR protein degradation via the lysosomal pathway. 

To further understand if the protective effect of CASK silencing in CHME3 is dependent on the enhanced EGFR activation, we generated EGFR silencing CHME3 cells. As shown in Figure 7F, EGFR silencing did not alter the CASK protein and mRNA expression. Moreover, unlike CASK silencing, EGFR silencing did not affect H_2_O_2-_induced cell death (Figure 7G). Additionally, gefitinib (5 μM), an EGFR tyrosine kinase inhibitor, also did not affect cell viability under H_2_O_2_ treatment in both shCTL and shCASK cells (Figure 7H). Taken together, these results indicate that the cell protective effect of CASK silencing is independent of EGFR. 

## 4. Discussion

Microglia are specialized macrophage-like cells that reside in the CNS. Although microglia are responsible for the preservation of brain homeostasis, they also trigger inflammatory responses and promote the development of neuronal disorders [38]. The deleterious effects of microglial activation in the progression of neuronal disorders have been extensively studied and firmly established. However, the mechanism of microglial death contributing to build up the neurotoxic environment remains unclear. CASK is a multidomain scaffold protein and is highly enriched in the human brain, especially within the neurons and microglia. A transcriptome study reveals that the alteration of CASK in the microglia contributes to the progression of neuropsychiatric disorders [39]. Despite this, the exact functional role of CASK in microglia is still unclarified. In this study, we used human CHME3 microglia, which possess the innate cellular functions of microglia like phagocytosis [40], expressing high chemokine receptors [41], and fast responses to infection [42] and hypoxia [43].

In this study, we first observed that CASK silencing in human CHME3 microglia significantly attenuates H_2_O_2_-induced cell death. Depending on the stress stimuli and cellular contexts, oxidative stress may lead to microglial apoptosis [44], parthanatos [45], or necroptosis [46,47]. Our study shows that H_2_O_2_-induced cell death is abrogated by the PARP-1 inhibitor olaparib, suggesting that the CHME3 cells undergo parthanatos under H_2_O_2_ treatment. The initiation of parthanatos cell death is induced by the DNA damage-mediated hyperactivation of PARP-1, which hydrolyses NAD^+^ to generate PAR polymers, then alters protein function via post-translational PARylation [48]. In common situations, PAR would translocate to the mitochondria, induce MMP loss [33,49,50], and promote mtROS generation [49]. Additionally, PARP-1 hyperactivation has been found to inhibit glycolysis [51], promote mitochondrial fission [31,45], and alter the mitochondrial respiratory chain [45], leading to bioenergetic failure and cell death. In our study, we observed that CASK silencing attenuates H_2_O_2_-induced PARylation, MMP loss, ROS production, and mitochondrial dysfunction (including mitochondrial fission, MMP loss, and OXPHOS inhibition). Using PARP-1 inhibitor olaparib, we confirm that the malfunction of mitochondria under H_2_O_2_ treatment is induced by PARP-1 activation. Moreover, CASK silencing protects cells by maintaining mitochondrial integrity and preserving higher cellular energy metabolism. Concomitantly, KEGG analysis also reveals that CASK knockout significantly alters mitochondrial oxidative phosphorylation [6]. 

The translocation of AIF from the mitochondria to the nucleus is a pivotal cell death cascade of parthanatos, as AIF forms a dimer with the MIF and the dimer complex translocates to the nucleus to cause chromatin condensation and DNA fragmentation. Surprisingly, we do not observe any translocation event of AIF into the nucleus under H_2_O_2_ administration, suggesting that an AIF-independent parthanatos cell death might be involved. Similarly, two studies demonstrate that H_2_O_2_ insult triggers parthanatos cell death in macrophages and ARPE-19 cells but does not induce AIF nuclear translocation. Moreover, siRNA-targeting AIF does not alter cell viability under H_2_O_2_ treatment [31,52]. Together with our result, we suggest that H_2_O_2_ is also capable of proceeding cell death through a non-canonical, AIF-independent parthanatos. More detailed signaling pathways and molecular mechanisms underlying this unique death mode warrant further investigation.

MAPKs (JNK, ERK, and p38), AKT, and AMPK have been shown to regulate parthanatos in different cell types under various stress conditions [23,32,33,34,35,36,53]. Consistently, we observed that all these signaling pathways except AMPK are activated in CHME3 cells under H_2_O_2_ treatment. We found that CASK silencing inhibits JNK and AMPK phosphorylation but enhances AKT phosphorylation under H_2_O_2_ treatment. Moreover, AKT inhibitor and AMPK activator abrogate the protective effect of CASK silencing. A recent study demonstrates the role of AKT in the regulation of parthanatos, as inhibition of AKT increases p53-SIRT6 expression and promotes ROS generation and subsequent parthanatos [36]. On the other hand, PARP-1 activation and subsequent ATP depletion lead to AMPK activation, which further enhances the nuclear translocation of AIF and promotes parthanatos [35]. Besides the AMPK-dependent promotion of parthanatos via AIF, AMPK-induced autophagic death [50] and mitochondrial dysfunction [54] have been demonstrated. In contrast, depending on the cellular context and cell types, AMPK activation exerts cell protection and maintains mitochondrial homeostasis [55,56]. In this study, we exclude the roles of AMPK-dependent autophagy and cell growth regulation in H_2_O_2_-induced microglial parthanatos. This notion is based on our observations of no autophagy induction by H_2_O_2_ and no effect of CASK silencing in regulating cell growth. Presently, we do not have mechanistic evidence underlying AMPK-mediated cell death in H_2_O_2_-treated shCASK microglia nor CASK-mediated regulation of AKT and AMPK. We will further explore these issues in the future. However, our current findings support previous observations on the opposite roles of AKT and AMPK in parthanatos.

In *C. elegans*, CASK regulates the basolateral membrane localization of EGFR, thereby promoting the proliferation and development of *C. elegans* [12]. In this study, we found that CASK silencing enhances the EGFR protein at both transcriptional and post-translational levels in CHME3 microglia. For the latter, we found that the EGFR protein is regulated by the lysosome but not the proteasome degradative pathway. Moreover, the inhibition of EGFR lysosomal degradation is involved in enhancing the EGFR protein level in CASK silencing cells. Although EGFR expression and activity are increased by CASK silencing, EGFR is not involved in the cell fate control as evidenced by the data using the EGFR inhibitor and EGFR silencing. To understand if the lysosomal degradation of EGFR results from autophagy, we measured autophagic flux and tested autophagic inhibitors bafilomycin A1 and 3-MA. However, we do not detect an increased LC3II/I protein ratio nor a change in the lysosomal mass under H_2_O_2_ treatment. Moreover, the extent of cell death is not altered by autophagic inhibitors in both control and CASK silencing cells. The lack of significant induction of autophagy might be due to the increased AKT activation caused by H_2_O_2_ and the decreased AMPK activation caused by CASK silencing. Another interesting finding in this study is to exclude the role of EGFR in H_2_O_2_-induced parthanatos. We speculate that EGFR activation is involved in regulating inflammation responses and cell migration in microglia as previously reported [25,26,27,28]. Moreover, because PARP-1 activation by UVB can induce ROS-dependent EGFR transactivation in keratinocytes [57], we will further address this issue in microglia in the future. 

In conclusion, we for the first time demonstrate the functional role of CASK in CHME3 microglia under oxidative stress. CASK silencing inhibits oxidative stress-induced AIF-independent parthanatos. CASK mediates PARP-1 activation and downstream death events such as ROS production, mitochondrial fission, MMP loss, and respiration inhibition. Moreover, CASK negatively regulates AKT but positively regulates AMPK, both of which oppositely control parthanatos. Currently it remains unclear how CASK regulates AKT and AMPK in microglia and the death pathway of parthanatos independent of AIF. These unclarified points are interesting and warrant for further investigation. Although CASK downregulates gene transcription and the protein stability of EGFR in CHME3 cells under oxidative stress, EGFR activity is not involved in regulating cell viability. Taken together, CASK plays a crucial role in oxidative stress-induced microglial cell death, and targeting CASK might be a potential therapeutic approach to relieve microglia-associated neuroinflammation and neurodegeneration. 

## Figures and Tables

**Table 1:** PCR primers.

Gene	Forward (5′ to 3′)	Reverse (3′ to 5′)	Gene Accession Number
β-Actin	CGG GGA CCT GAC TGA CTA CC	AGG AAG GCT GGA AGA GTG C	NG_007992
CASK	TTG AAA TCG TAA AGC GAG CTG A	CAG TAG CGT AGA GCT TCC AGT A	NG_016754
EGFR	GAC CTC CAT GCC TTT GAG AA	GCT GAC GAC TGC AAG AGA AA	NG_007726

## Data Availability

Data are contained within the article and Supplementary Materials.

## Supplementary Materials

The following supporting information can be downloaded at https://www.mdpi.com/article/10.3390/antiox13030343/s1, Figure S1: H2O2 does not induce autophagy

## Abbreviations

AIF	Apoptosis-inducing factor
AKT	Protein kinase B
AMPK	Adenosine monophosphate-activated protein kinase
ATP	Adenosine triphosphate
BafA1	Bafilomycin A1
CASK	Calcium/calmodulin-dependent serine protein kinase
CHX	Cycloheximide
CNS	Central nervous system
DAPI	4′,6-Diamidino-2-phenylindole
DHE	Dihydroethidium
DMEM	Dulbecco’s Modified Eagle Medium
EGFR	Epidermal growth factor receptor
ERK	Extracellular signal-regulated kinase
FBS	Fetal bovine serum
FCCP	Carbonyl cyanide-p-trifluoromethoxyphenylhydrazone
H_2_O_2_	Hydrogen peroxide
JNK	c-Jun N-terminal kinase
LC3	Microtubule-associated protein light chain 3
LPS	Lipopolysaccharide
3-MA	3-Methyladenine
MAPKs	Mitogen-activated protein kinases
MIF	Macrophage migration inhibitory factor
MMP	Mitochondrial membrane potential
mtROS	Mitochondrial ROS
NAD	Nicotinamide adenine dinucleotide
Nec-1	Necrostatin-1
OCR	Oxygen consumption rate
PAR	Poly(ADP-ribose)
PARP-1	Poly (ADP-ribose) polymerase 1
PBS	Phosphate buffered saline
PI	Propidium iodide
ROS	Reactive oxygen species
RT	Reverse transcription
RT-PCR	Real-time polymerase chain reaction
shRNA	Short hairpin RNA
TOM20	Translocase of outer mitochondrial membrane 20
zVAD-FMK	Carbobenzoxy-valyl-alanyl-aspartyl-[O-methyl]-fluoromethylketone
