# Supplementary figures and images for: CASK Mediates Oxidative Stress-Induced Microglial Apoptosis-Inducing Factor-Independent Parthanatos Cell Death via Promoting PARP-1 Hyperactivation and Mitochondrial Dysfunction
Source: Antioxidants (Basel). 2024 Mar 13;13(3):343. doi: 10.3390/antiox13030343 (PMC10968130; doi:10.3390/antiox13030343)

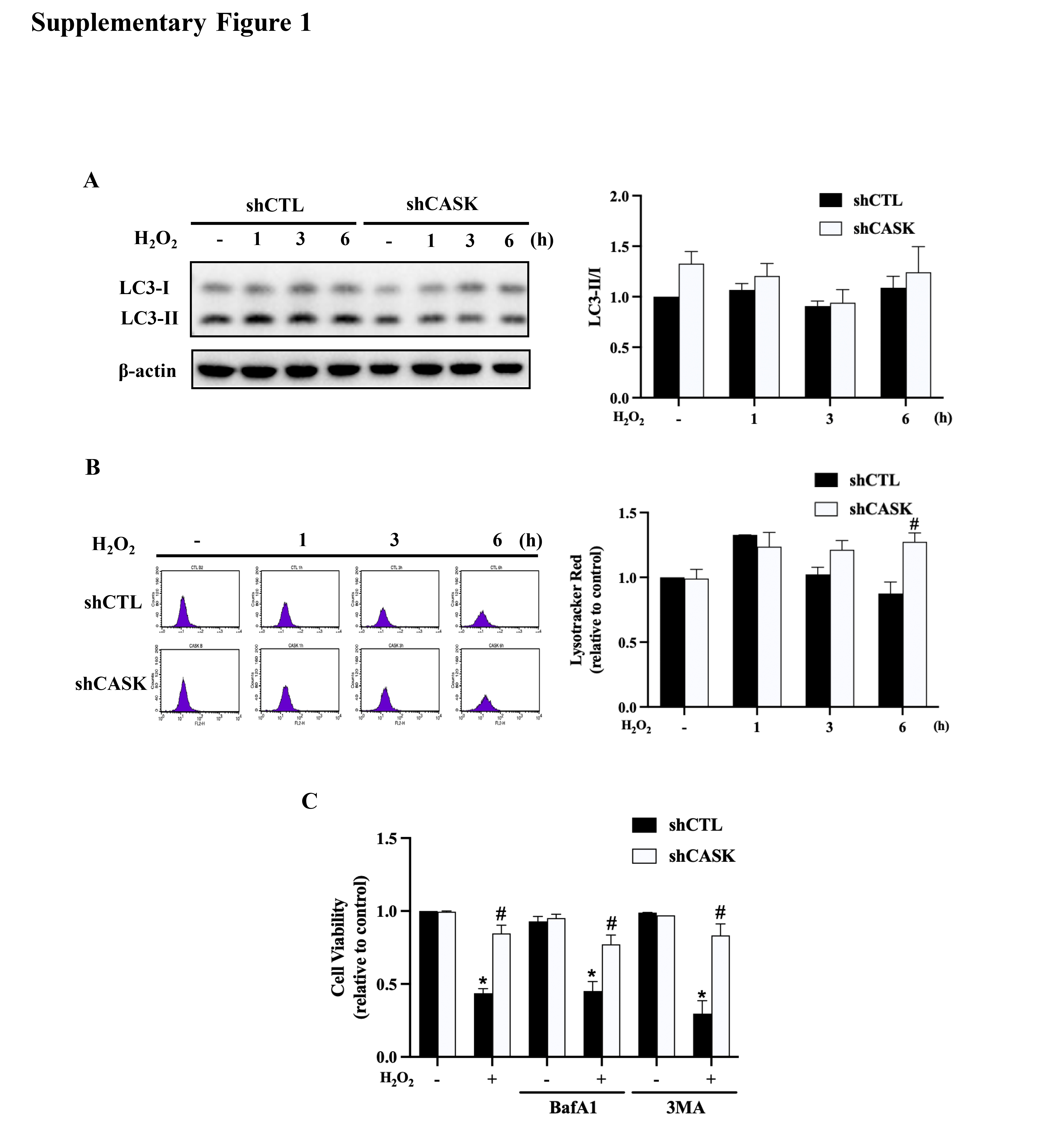

Supplement: Supplementary file 1 [file antioxidants-13-00343-s001.zip › antioxidants-2787022-supplementary.jpg]
